# Differences in Immune Defense Evasion of Selected Inbred Lines of *Heterorhabditis Bacteriophora* in Two White Grub Species

**DOI:** 10.3390/insects3020378

**Published:** 2012-03-23

**Authors:** Ruisheng An, Marcio Voss, Ganpati B. Jagdale, Parwinder S. Grewal

**Affiliations:** 1 Department of Entomology, Ohio State University, 1680 Madison Ave, Wooster, OH 44691, USA; E-Mail: an.48@osu.edu; 2 Embrapa Trigo, Passo Fundo, Rio Grande do Sul 99001-970, Brazil; E-Mail: marciov@cnpt.embrapa.br; 3 Department of Plant Pathology, University of Georgia, Athens, GA 30605, USA; E-Mail: gbjagdal@uga.edu

**Keywords:** entomopathogenic nematodes, *Heterorhabditis bacteriophora*, inbred lines, white grubs, immune defense

## Abstract

We determined virulence of seven *Heterorhabditis bacteriophora* strain GPS11 inbred lines possessing superior infective juvenile longevity, and heat and ultra violet radiation tolerance against white grubs *Popillia japonica* and *Cyclocephala borealis*. At 1 and 2 weeks after treatment, inbred line A2 was significantly more virulent towards *P. japonica* compared to the parent strain GPS11 and inbred lines A7, A8, A12 and A21; and line A2 caused significantly higher *C. borealis* mortality than lines A6 and A12. Penetration, encapsulation and survival of two inbred lines, A2 and A12, that showed the highest and lowest virulence against both grub species were then assessed. There were no differences between the two lines for the total number of nematodes penetrated in either *P. japonica* or *C. borealis* within the first 24 h, but a significantly higher percentage of penetrated nematodes were alive in line A2 compared to the line A12 in both grub species. *P. japonica* immune response over time to hemocoel-injected nematodes of A2, A12 and the parent strain was further investigated. While all injected nematodes were encapsulated at 6 h post injection, non-encapsulated living nematodes were detected at 12 and 24 h post injection, showing the breakage out of encapsulation. A higher percentage of non-encapsulated living nematodes and a lower percentage of dead nematodes were found in line A2 as compared to the line A12 after 12 h post injection. These data suggest that virulence differences in the studied *H. bacteriophora* inbred lines are not due to differences in nematode penetration or recognition by the grub immune system, but are related to the ability of the infective juveniles to break out of encapsulation.

## 1. Introduction

Entomopathogenic nematodes (EPNs) *Steinernema* and *Heterorhabditis* are lethal insect parasites. The nematode infective juveniles (IJs) penetrate the insect host generally through natural body openings and release mutualistic bacteria (*Xenorhabdus* spp for *Steinernema* and *Photorhabdus* spp for *Heterorhabditis*) in the hemocoel. The nematodes and the multiplying bacteria in the hemocoel produce virulence factors resulting in death of the host within 2–3 days after infection [1,2]. Developing nematodes feed on the bacteria and disintegrated host tissues, produce 1–3 generations, and when the food resources are depleted, nematodes emerge as IJs to seek new hosts.

While EPNs are effective biological control agents of many soil-dwelling insect pests [3], their use against white grubs is still limited. As white grubs are an important pest complex requiring effective control [4], interest in the commercial development of more potent nematode strains is high. Previous studies [5,6] show that, from among the many strains of *Heterorhabditis bacteriophora,* the GPS11 strain possesses superior traits, including higher virulence against several white grub species, longer longevity of IJs, and higher stress tolerance compared to the commercial strains. However, it has been reported that desirable traits in EPNs may deteriorate over time due to inadvertent laboratory selection [7,8,9].

Considering that inbreeding can be used to identify and fix desirable traits [9,10], we established 35 inbred lines in the GPS11 strain of *H. bacteriophora* and identified lines with superior IJ longevity, heat and ultra violet radiation (UV) tolerance, and virulence against the wax moth *Galleria mellonella* larvae [11]. In this study, we tested seven inbred lines possessing these fixed superior traits for differences in virulence against two white grub species, *Popillia japonica* and *Cyclocephala borealis*, to identify a “super” inbred line with multiple desirable traits fixed against potential genetic deterioration. We also determined if the differences in the virulence of inbred lines is due to the differences in their penetration rate or subsequent survival in the two grub species. As combating the host immune response plays an important role in successful infectivity of EPNs, we further investigated the fate of the hemocoel-injected nematodes in *P. japonica*, overtime. We hypothesized that the differences in the virulence of these inbred lines is related to the ability of IJs to defend against the host immune response, but not to the rate of their penetration into the host.

## 2. Experimental Section

### 2.1. Sources of White Grubs

The second instar * P. japonica* grubs were obtained by caging the adult beetles in PVC cylinders (20 cm diam. × 15 cm tall) dug into a turfgrass lawn following procedures described by Klein *et al*. [4]. The beetles were caught early in the summer using trécé catch can traps (Trécé Inc., Adair, OK, USA) containing a feeding lure. When the grubs had developed to approximately 50% first and 50% second instars, the area within each cylinder was dug and second instar grubs were collected from the rhizospere of turfgrass. All the collected grubs were then stored in plastic trays containing field soil at 10 °C until used in the assays. The third instar *C. borealis* grubs used in this study were collected from the naturally infested turfgrass areas on different golf courses in Wayne County, Ohio and stored as described above until used.

### 2.2. Source of *H. bacteriophora* Inbred Lines

Inbred lines used in this study were established from the GPS11 strain of *H. bacteriophora*, which was originally isolated from a single larva of *C. borealis* in 1998 at Atwood Lake Golf course, Dellroy, Ohio, USA [5]. Each last instar *Galleria mellonella* larva was exposed to a single IJ in the one-on-one sand-well method as previously described [12]. As *H. bacteriophora* IJs always develop into self-reproducing hermaphrodites [13], it is possible to establish inbred lines from single IJs. Each infected insect cadaver was then placed on a separate White trap [14] and next generation IJs were collected from the cadavers as individual inbred lines. This procedure was repeated 10 times (≈20 to 30 generations), resulting in a total of 35 inbred lines [11]. Out of these, seven inbred lines designated as A2, A6, A7, A8, A12, A18 and A21 were selected for this study as they exhibited superior IJ longevity, heat and UV tolerance, and virulence against the wax moth *G. mellonella* larvae as determined previously [11]. All the lines were stored in tissue culture flasks at 10 °C until used in the tests. Before the tests, all the lines were recycled once through the wax moth *G. mellonella* larvae at 25 °C [15]. Similarly, cryopreserved IJs of GPS11 (parent strain) were removed from liquid nitrogen storage and recycled once through *G. mellonella* larvae at 25 °C. Only IJs emerging from host cadavers within the first 2–5 days were used in all tests.

### 2.3. Virulence of Inbred Lines against *P. japonica* and *C. borealis*

Separate bioassays were conducted for *P. japonica* and *C. borealis* to compare the virulence of inbred lines and the parental strain GPS11 of *H. bacteriophora*. The virulence of nematodes was tested in 30 mL plastic cups containing 29 g autoclaved Wooster silt loam soil. The soil was first passed through a 4 mm mesh sieve, and then transferred into each cup, and soil moisture was adjusted to 85% field capacity by adding the required amount of water. One *P. japonica* or one *C. borealis* was placed on the surface of the soil in each cup, lids were replaced, and the grubs were allowed to acclimate overnight at room temperature 25 °C. The grubs that did not burrow into the soil within 12 h were replaced. Then 100 µL nematode suspension containing 100 IJs was added to the surface of the soil in each cup. As a control treatment, each cup received 100 µL of water without nematodes. All the cups were arranged in a randomized block design with four replications and each replication contained 10 cups (n = 10 grubs). All cups were incubated at 25 °C. Both bioassays were repeated using new batches of grubs and freshly cultured IJs. Grub mortality was recorded twice at 1 and 2 weeks after treatment (WAT). Percentage mortality data were corrected using Abbott’s formula [16]. The corrected percent mortality data from both bioassays were pooled, as there were no differences in the two tests based on one-way ANOVA. The corrected percentage mortality data were transformed to arcsine and nematode penetration data were normalized by transforming to log+1 prior to ANOVA. Significant differences between treatments were determined using Tukey’s test at *P* < 0.05.

### 2.4. Penetration, Encapsulation and Survival of Inbred Lines in *P. japonica and C. borealis*

Two bioassays, one on *P. japonica* and one on *C. borealis*, were conducted to compare the penetration, encapsulation escape and survival capacities of inbred lines that showed the highest and lowest virulence against both grub species. The parental strain GPS11 was also included in the tests. Tests were conducted following the previously described method [5]. Briefly, 30 g autoclaved fine sand was transferred into 30 mL plastic cups and the moisture was adjusted to 8% by adding the required amount of water. The sand was used for its ease in being washing off the grub body as described below. Then, a 100 µL suspension, containing 200 IJs of A2, A12 or GPS11 was added to the surface of sand in each cup and the lid was replaced. As a control treatment, each cup received 100 µL of water without nematodes. A total of 8 such cups were prepared for each treatment and each cup was considered as a separate replication. One actively moving *P. japonica* or *C. borealis* grub was introduced in the center of each cup by making a hole in the sand with a glass rod, and lids were replaced. All the cups were then arranged in a randomized block design at 25 °C. After 24 h of incubation, each grub was removed from the cup and washed in a 50 mL beaker containing 10 mL tap water to remove any sand particles and nematodes adhering to the body. Only 24 h incubation period was used to capture initial host response against the leading nematode penetrants. Each grub was dissected in a 5 cm diameter petri dish containing 10 mL of tap water. The grub was decapitated, the ventral tip of the abdomen was partially excised and a ventral cut was made along the body. The digestive system was then removed. The number of nematodes penetrated was recorded as numbers of dead, alive and encapsulated nematodes using a stereomicroscope. The dark-colored and blood cell-attached nematodes were recorded as encapsulated. Only the *P. japonica* bioassay was repeated using a new batch of grubs and freshly cultured nematodes because of the lack of availability of *C. borealis* grubs. The penetration data for *P. japonica* from both bioassays were pooled, as there were no differences in the two tests based on one-way ANOVA. The percentage data were transformed to arcsine and normalized by transforming to log+1 prior to ANOVA. Significant differences between treatments were determined using Tukey’s test at P < 0.05.

### 2.5. Temporal Interactions of Inbred Lines with Immune Response of *P. japonica*

We compared the interactions of two inbred lines A2, A12 and the parent strain GPS11 with immune response of *P. japonica* grubs over time. The IJs of lines A2, A12 and GPS11 were directly injected into the hemocoel of *P. japonica* grub from the foreleg. In order to obtain reproducible injection of nematodes, we used disposable sterile pipet tips with microcapillary (1–200 µL). The pipet tip was cut off using a sterile scissor to obtain a sharp pointed tip, and the uptake and release of nematodes was verified under the dissecting microscope by observing nematodes in the plastic capillary. Prior to injection, the grubs were washed with sterile tap water and cleaned with 70% ethanol at the injection site. About 20 IJs with 10 µL sterile water were injected laterally into the base of the first leg. Grubs in the control treatment were injected with hot water killed IJs. If the digestive system was damaged during injection, the grub was discarded. For each nematode line, there were three time intervals (6, 12, and 24 h post injection) at which injected grubs were dissected to observe the fate of the nematodes. The injected grubs were incubated at 25 °C. At each time interval, 10 *P. japonica* grubs for each treatment were decapitated, the ventral tip of the abdomen was partially excised and a ventral cut was made along the body. The digestive system was then removed. Water was flushed through the open hemocoel and examined for nematodes under a dissecting microscope. Other tissues were then carefully dissected and examined. The number of encapsulated living, non-encapsulated living and dead nematodes was recorded. The experiment was repeated once, and data indicated no differences in two experiments by one-way ANOVA and were pooled. The percentages of nematodes in three categories, relative to the total number injected, were then arcsine transformed and subjected to one-way ANOVA. 

## 3. Results

### 3.1. Virulence of Inbred Lines against *P. japonica and C. borealis*

**Figure 1:**
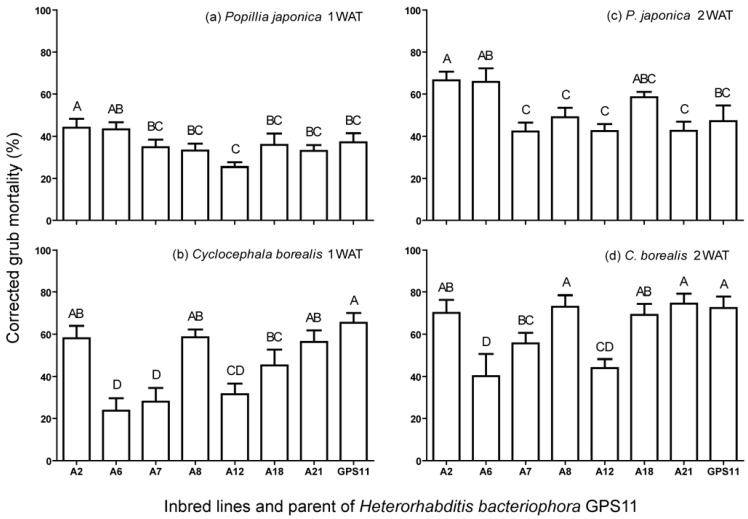
Mean percentage mortality of *Popillia japonica* and *Cyclocephala borealis* grubs caused by inbred lines and parent of *H. bacteriophora* GPS11 strain 1 and 2 weeks after treatment (WAT). Bars (mean ± SEM) with same letter(s) are not significantly different according to *P* < 0.05 (Tukey’s HSD test).

Overall there were significant differences in virulence of inbred lines against *P. japonica* (*F* = 3.04; df = 3, 15; *P* < 0.05) and *C. borealis* (*F* = 13.30; df = 7, 15; *P* < 0.05). At 1 week after treatment (WAT), line A2 caused more mortality of *P. japonica* than most of the other lines (A7, A8, A12, A18, A21) and even the parent strain GPS11 (Figure 1). Similarly, at 2 WAT, line A2 caused significantly higher *P. japonica* mortality than A7, A8, A12, A21 and the parent strain GPS11 (Figure 1). Comparatively, line A12 caused lowest *P. japonica* mortality (Figure 1). In case of *C. borealis*, the inbred lines A2, A8 and A21, and the parent strain caused significantly higher grub mortality than A6, A7 and A12 at 1 WAT (Figure 1). At 2 WAT, the inbred lines A2, A8, A18 and A21 caused significantly higher *C. borealis* mortality than A6 and A12 but it was not different from the parent strain (Figure 1). Taken together, line A2 caused significantly higher mortality than A12 in both *P. japonica* and *C. borealis* (Figure 1).

### 3.2. Penetration, Encapsulation and Survival of Inbred Lines in *P. japonica and C. borealis*

**Figure 2:**
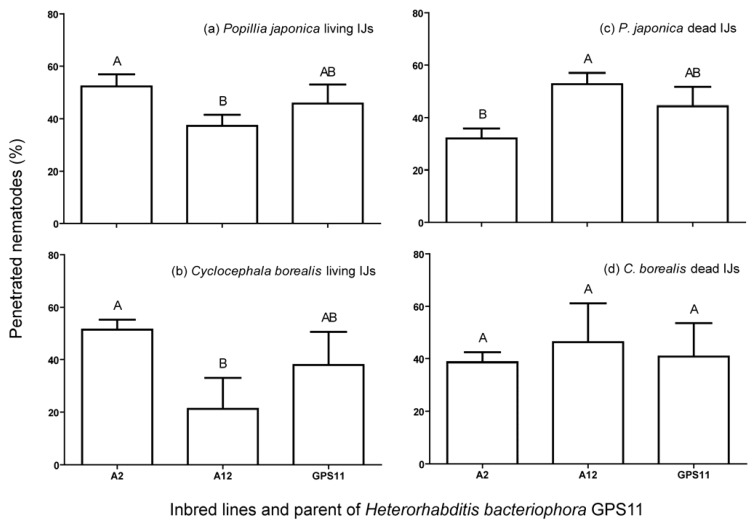
Mean percentage of live and dead infective juveniles of inbred lines, A2 and A12 and parent GPS11 strain of *Heterorhabditis bacteriophora* found in *Popillia japonica* and *Cyclocephala borealis* grubs 24 h after incubation. Bars (mean ± SEM) with same letter(s) are not significantly different according to *P* < 0.05 (Tukey’s HSD test).

Overall penetration of *H. bacteriophora* inbred lines differed with white grub species (*F* = 1.85; df = 7, 5; *P* < 0.05). Total penetration ranged from 3 to 6% in *P. japonica* and 2.18 to 2.25% in *C. borealis* during the first 24 h, and specifically line A12 showed significantly higher penetration in *P. japonica* than *C. borealis*. While the total number of nematodes penetrated in *P. japonica* (*F* = 1.92; df = 15, 2; *P* < 0.05) and *C. borealis* (*F* = 0.37; df = 7, 5; *P* < 0.05) grubs did not differ significantly between the inbred lines, there were significant differences in the survival of nematodes between inbred lines in both grub species (Figure 2). Of the total number of nematodes that penetrated, significantly higher percentages were found alive in case of line A2 compared to A12 in both *P. japonica* and *C. borealis* (*F* = 2.20; df = 15, 5; *P* < 0.05 for *P. japonica* and *F* = 1.13; df = 7, 5; *P* < 0.05 for *C. borealis*) (Figure 2). The percentage of dead nematodes was significantly higher (*F* = 2.20; df = 15, 5; *P* < 0.05) in line A12 compared to A2 in case of *P. japonica* only. None of the lines differed from the parent strain GPS11 in either survival or encapsulation in either grub species.

### 3.3. Temporal Interactions of Inbred Lines with Immune Response of *P. japonica*

Almost 100% of the nematodes in each treatment were encapsulated at 6 h post hemocoel-injection, and no non-encapsulated and dead nematodes were observed at this time. Nematode treatment influenced the percentages of nematodes encapsulated at 24 h (F = 10.83; df = 2, 57; P = 0.000), non-encapsulated living nematodes at both 12 h (F = 5.42; df = 2, 57; P = 0.007) and 24 h (F = 38.17; df = 2, 57; P = 0.000), and dead nematodes at 24 h (F = 12.89; df = 2, 57; P = 0.000) post infection. Compared to the A12 line, the A2 line had a higher percentage of non-encapsulated living, a lower percentage of dead at both 12 and 24 h post nematode injection, and lower percentage of encapsulated nematodes (including both alive and dead nematodes) at 24 h post injection. In contrast to line A2, line A12 had the least non-encapsulated living (P < 0.05) at both 12 and 24 h post injection, and the most dead (P < 0.05) at 24 h post injection IJs.

## 4. Discussion and Conclusions

This study demonstrates a variation in virulence of inbred lines established from the GPS11 strain of *H. bacteriophora* against both *P. japonica* and *C. borealis* grubs. The inbred line A2 showed significantly higher virulence than A12 against both *P. japonica* and *C. borealis*. This is consistent with Glazer *et al*. [17] who reported the virulence variation in inbred lines of *H. bacteriophora* HP88 against the laboratory host *G. mellonella* and Bai *et al*. [9], who detected differences in virulence among inbred lines of *H. bacteriophora* against Diaprepes root weevil, *Diaprepes abbreviatus*. Remarkably, our results support the hypothesis that there is genetic diversity in virulence of *H. bacteriophora* against economically important pests, because the inbred lines would have performed similarly if this trait had no genetic basis [7]. Together, it confirms that selective breeding may be a legitimate approach for improving virulence of entomopathogenic nematodes.

Our results indicate that the differences in virulence of *H. bacteriophora* inbred lines are not due to differences in their host penetration rate, but related to their ability to survive in the white grubs. We found a higher portion of living nematodes for line A2 than for A12 in both *P. japonica* and *C. borealis,* although the penetration rate was not significantly different between these two lines. Dissections of nematode injected *P. japonica* grubs over time revealed that the initial *P. japonica* immune response did not differ against the two *H. bacteriophora* lines and parent strain GPS11, but that the number of nematodes breaking out of encapsulation differed among the treatments. IJs of all the inbred lines were equally recognized by hemocytes 6 h post injection and the percentage of encapsulated nematodes did not differ between inbred lines 12 h post injection. However, percentages of free-moving and dead nematodes differed between inbred lines 12 h post injection, suggesting that the differences in the virulence of inbred lines are due to the differences in their ability to get out of the encapsulation. Although this phenomenon has been observed in previous studies in which 10% of *H. bacteriophora* HP88 freed themselves from encapsulation in *P. japonica* larvae after 24 h [18,19], this study shows that breaking out of encapsulation is an important mechanism of defense of *H. bacteriophora* IJs in the white grub hemocoel.

We found a higher proportion of dead nematodes in the white grubs in the exposure bioassay as compared to the direct injection experiment. This may be due to the slow rate of nematode invasion in the exposure bioassay, allowing more time for the grub’s immune response to build up and kill the entering nematodes. In agreement, during the natural route of penetration, some nematodes may have been killed in the gut fluid due to high pH and proteases, or their cuticle may have been denatured before entry into the hemocoel [19,20]. This may have been the case, as we did not separately record nematodes in the gut and hemocoel in the exposure bioassay experiments.

**Figure 3:**
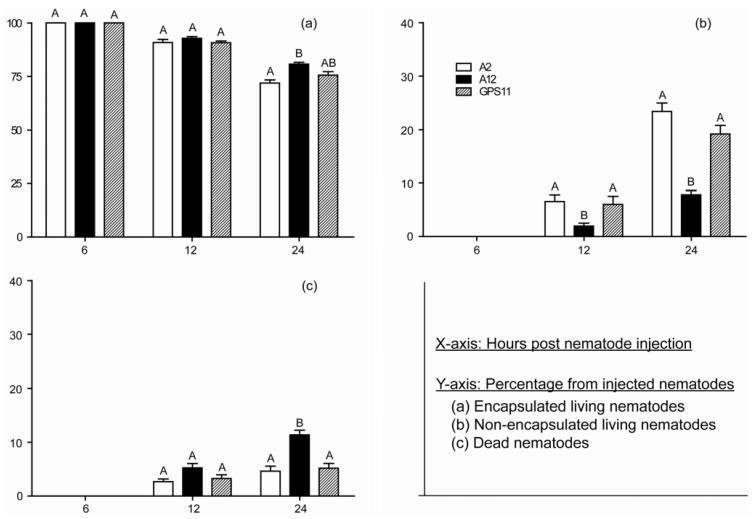
Percentages of encapsulated living, non-encapsulated living, and dead *Heterorhabditis bacteriophora* nematodes in *Popillia japonica* grubs at 6, 12 and 24 h post hemocoel-injection with infective juvenile nematodes of line A2, line A12 and GPS11 strain. The percentages (mean ± SEM) of encapsulated living, non-encapsulated living and dead nematodes were compared separately at each time interval. The same letter indicates no statistical difference at P = 0.05 (Tukey’s HSD test).

While previous studies have reported differences in the susceptibility of *Heterorhabditis* and *Steinernema* species to immune response of white grubs [5,18,19,21], our results suggest that the mechanism of defense against the host immune response may be different between the heterorhabditids and steinernematids. In *G. mellonella* larvae, *Steinernema carpocapsae* is not recognized as being foreign and is not encapsulated in the hemocoel [22]. Although recognized and encapsulated by hemocytes of the Japanese beetle *P. japonica*, *Steinernema glaseri* IJs were found to be all free of hemocytes or their products 24 h after injection whereas less than 30% of *H. bacteriophora* were free (see Figure 3). Brivio *et al*. [20] have shown that *Steinernema feltiae* suppresses activity of prophenoloxidase, known as proPO enzymatic cascade, to avoid being encapsulated. Therefore, we assume that heterorhabditids and steinernematids may resist the insect host immune response by breaking out of and suppressing encapsulation, respectively.

The bacteria released by EPNs are considered the major cause of insect mortality as previous studies have found that axenic *Heterorhabditis* nematodes can efficiently infect insects, but cause much less (or even no) insect mortality compared to the nematode-bacteria complex or the bacteria alone [23,24,25]. However, nematodes do not immediately release their mutualistic bacteria upon entry into a host [19,26], which allows an opportunity for rapid host immune response to be mounted against the invading nematodes, resulting in encapsulation and sometimes killing of the nematodes. We found that 100% of the invading nematodes were encapsulated at 6 h post hemocoel-injection, confirming that the delay in bacterial release provides an opportunity for rapid host immune response to encapsulate and kill nematodes. Some nematodes escaping from encapsulation were observed at 12 h post injection, while very few bacteria are released into the grub hemolymph within the first 24 h period after injection [27]. Thus, the higher virulence of A2 may be attributed to the higher proportion of nematodes breaking out of encapsulation but not due to differences in the rate of bacterial release. However, the role of bacteria in this process still needs to be further investigated.

It has been previously reported that inbred lines of *H. bacteriophora* can be superior to the parent strain with respect to desiccation tolerance, heat resistance and host finding [11,17,28]. This may be due to a heterosis effect in which positive effects occur in the progeny of crosses of homozygous individuals carrying different alleles [28]. Our findings on differences in virulence of inbred lines against white grub species further emphasize the importance of establishing superior inbred lines for use in the biological control of insect pests. While decline in host-seeking ability, virulence, reproduction potential, storage ability and stress tolerance (desiccation, heat and ultra violet radiation) of *H. bacteriophora* has been observed after serial culturing in *G. mellonella*[7,8,9], use of inbred lines may be preferable over the wild type strains due to the potential for fixing of desirable traits in the inbred lines compared to heterozygous wild type strains [9,10]. Thus, line A2 can be considered as a candidate for potential commercialization compared with the parent strain because of its superior and fixed virulence against both *P. japonica* and *C. borealis* and its superior IJ longevity, and heat and UV tolerance. Also, the lines A2 and A12 can serve as research tools for in-depth investigation into the mechanisms of nematode defense against the host immune response.
